# Organochemical Characterization of Peat Reveals Decomposition of Specific Hemicellulose Structures as the Main Cause of Organic Matter Loss in the Acrotelm

**DOI:** 10.1021/acs.est.2c03513

**Published:** 2022-11-18

**Authors:** Henrik Serk, Mats B. Nilsson, João Figueira, Jan Paul Krüger, Jens Leifeld, Christine Alewell, Jürgen Schleucher

**Affiliations:** † Department of Medical Biochemistry and Biophysics, Umeå University, SE-90187 Umeå, Sweden; ‡ Department of Forest Ecology and Management, Swedish University of Agricultural Sciences, SE-90183 Umeå, Sweden; § Department of Chemistry, SciLife Lab, Umeå University, SE-90187 Umeå, Sweden; ∥ UDATA GmbH − Umwelt und Bildung, Hindenburgstrasse 1, 67433 Neustadt an der Weinstraße, Germany; ⊥ Departement Umweltgeowissenschaften, Universität Basel, Bernoullistrasse 30, CH-4056 Basel, Switzerland; # Agroscope, Climate and Agriculture Group, Reckenholzstrasse 191, CH-8046 Zurich, Switzerland

**Keywords:** 2D NMR, hemicellulose, cellulose, peat, organic matter, acrotelm

## Abstract

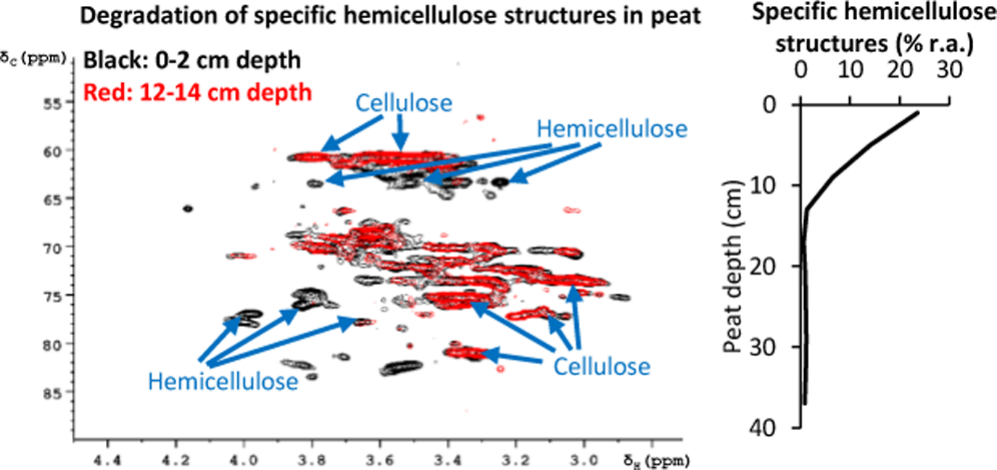

Peatlands store carbon in the form of dead organic residues. Climate change and human impact impose risks on the sustainability of the peatlands carbon balance due to increased peat decomposition. Here, we investigated molecular changes in the upper peat layers (0–40 cm), inferred from high-resolution vertical depth profiles, from a boreal peatland using two-dimensional ^1^H–^13^C nuclear magnetic resonance (NMR) spectroscopy, and comparison to δ^13^C, δ^15^N, and carbon and nitrogen content. Effects of hydrological conditions were investigated at respective sites: natural moist, drainage ditch, and natural dry. The molecular characterization revealed preferential degradation of specific side-chain linkages of xylan-type hemicelluloses within 0–14 cm at all sites, indicating organic matter losses up to 25%. In contrast, the xylan backbone, galactomannan-type hemicelluloses, and cellulose were more resistant to degradation and accumulated at the natural moist and drainage site. δ^13^C, δ^15^N, and carbon and nitrogen content did not correlate with specific hemicellulose structures but reflected changes in total carbohydrates. Our analysis provides novel insights into peat carbohydrate decomposition and indicates substantial organic matter losses in the acrotelm due to the degradation of specific hemicellulose structures. This suggests that variations in hemicellulose content and structure influence peat stability, which may have important implications with respect to climate change.

## Abstract 2

Molecular changes in peat are not well understood. This study reveals substantial organic matter losses in the acrotelm caused by the degradation of specific hemicellulose structures.

## 1 Introduction

Peatlands are an essential part of the global carbon (C) pool as more than 30% of global soil-C is stored in boreal peatlands.^1^ The ability of peatlands to store large amounts of C is attributed to low decomposition rates of organic matter (OM) compared to other soils. This slow decomposition arises from specific chemical properties of *Sphagnum* peat mosses, relatively high water table levels that generate anoxic conditions, and the generally low temperatures in boreal peatlands.^2−6^ Undisturbed peatlands usually serve as C sinks where net photosynthetic C uptake by *Sphagnum* mosses exceeds peat decomposition rates.^2,6−8^ However, peat decomposition depends on the availability of oxygen: aerobic decomposition of OM mostly results in the production of CO_2_, whereas anaerobic conditions are favorable for CH_4_ production. Aerobic C losses are considered the major source of C loss and are generally greater than anaerobic decomposition processes.^9^ High water table levels limit oxygen diffusion; therefore, the water table level plays an important role in defining the stage of peat decomposition. Climate change increases the frequency and persistency of drought events, leading to lower water table depths and therefore increased peat decomposition.^10,11^ In addition, human activities, such as drainage, increase aeration of peat, which often leads to increased C losses.^12,13^

Saprotrophic respiration rates from peat OM decomposition are strongly correlated with the proportion of polysaccharides.^14−17^ In the peat depth column, polysaccharides are preferentially degraded, whereas aromatic compounds such as lignin and aliphatic compounds are relatively enriched.^18,19^ Peat composition is also affected by vegetational and/or hydrological changes, which primarily influence the composition of lignin and other aromatic compounds.^19−21^ Although the general trend in polysaccharides, aromatics, and aliphatics is relatively well understood, little is known about the behavior of specific molecular substructures within these compound groups.

The aim of the present study is to elucidate changes in molecular substructures during peat composition in relation to ecophysiological constrains as well as potential relationships with common isotopic and elemental proxies. This is a specific manifestation of the general question if substructures of OM are labile or recalcitrant, or if their properties must be evaluated in the ecological context.^22,23^ Conventional methods such as pyrolysis-GC-MS, FT-IR spectroscopy, and one-dimensional NMR spectroscopy have shortcomings in studying this on the required molecular level. The reason is that these methods are either destructive or lack the resolution to distinguish between linkages of specific compounds. Recently, Soucémarianadin et al.^24^ showed that two-dimensional ^1^H–^13^C liquid-state NMR spectroscopy can be applied to soil OM, allowing nondestructive detection of specific molecular moieties, i.e., molecular substructures, with high resolution. Although this method has been extensively applied to study cell wall structures and composition of higher plants,^25−27^ it has only recently been used to study molecular structures of peat.^24^

In addition to analytical methods, various proxies have been proposed for in predicting changes in peat OM decomposition including the C/N ratio,^28^ stable carbon and nitrogen isotopes (δ^13^C and δ^15^N, respectively),^29,30^ as well as biomarkers.^31,32^ The δ^13^C signature of surface peat vegetation is modulated by fractionation during photosynthetic C uptake.^33−35^ During peat decomposition, δ^13^C changes due to preferential breakdown of polysaccharides and the accumulation of isotopically lighter compounds such as lignin, which increases δ^13^C of the peat.^22^ Oxygen availability may play an important role as well since decomposition processes differ between aerobic and anaerobic conditions.^29^

The δ^15^N signature of peat displays a distinct depth pattern, depending on hydrological status, triggering differing microbial community composition.^30^ In the acrotelm of nutrient-poor fens, this pattern may further depend on interactions between plant roots and mycorrhiza, as well as fungi-dominated decomposition.^30,31,36−38^ Recently, it has been proposed that microbial decomposition processes overcome the effects of mycorrhizal symbiosis.^39^ In this context, it has also been found that δ^15^N reflects changes in microbial metabolic processes.^31^

The C/N ratio has been proposed to indicate peat decomposition.^28,29^ In contrast to mineral soils, peat has a high C content with an average of 49% for northern peatlands,^1^ due to low decomposition rates. In general, C/N ratios of peat decrease with depth in the acrotelm, as more C is lost relatively to N during decomposition processes. However, the C/N ratio may be influenced by changes in vegetation, N deposition rates, and hydrological/moisture conditions.^40−42^ In summary, it is not well understood how isotopic and elemental signatures reflect molecular changes in structural components of peat OM, specifically with respect to differences in moisture and drainage.

Here, we used ^1^H–^13^C liquid-state NMR spectroscopy to identify changes in molecular substructures during peat decomposition in the acrotelm and upper mesotelm (0–40 cm) of high-resolution peat depth profiles from three different sites of a peatland in northern Sweden (Degerö Stormyr), with different hydrological conditions: natural moist, natural dry, and a drainage ditch. The acrotelm and upper mesotelm are particularly relevant because most C losses occur here and the coupling of these upper layers to the bog vegetation may cause feedbacks to the bog vegetation. Potential relationships with common isotopic and elemental proxies were identified by comparing depth-dependent changes in molecular structures to the peat decomposition indicators (δ^13^C, δ^15^N, C and N content, and C/N ratio). To our knowledge, this is the first study that uses liquid-state 2D NMR to characterize changes in molecular structures in peat depth profiles.

## 2 Materials and Methods

### 2.1 Peat Core Sampling

**Figure 1:**
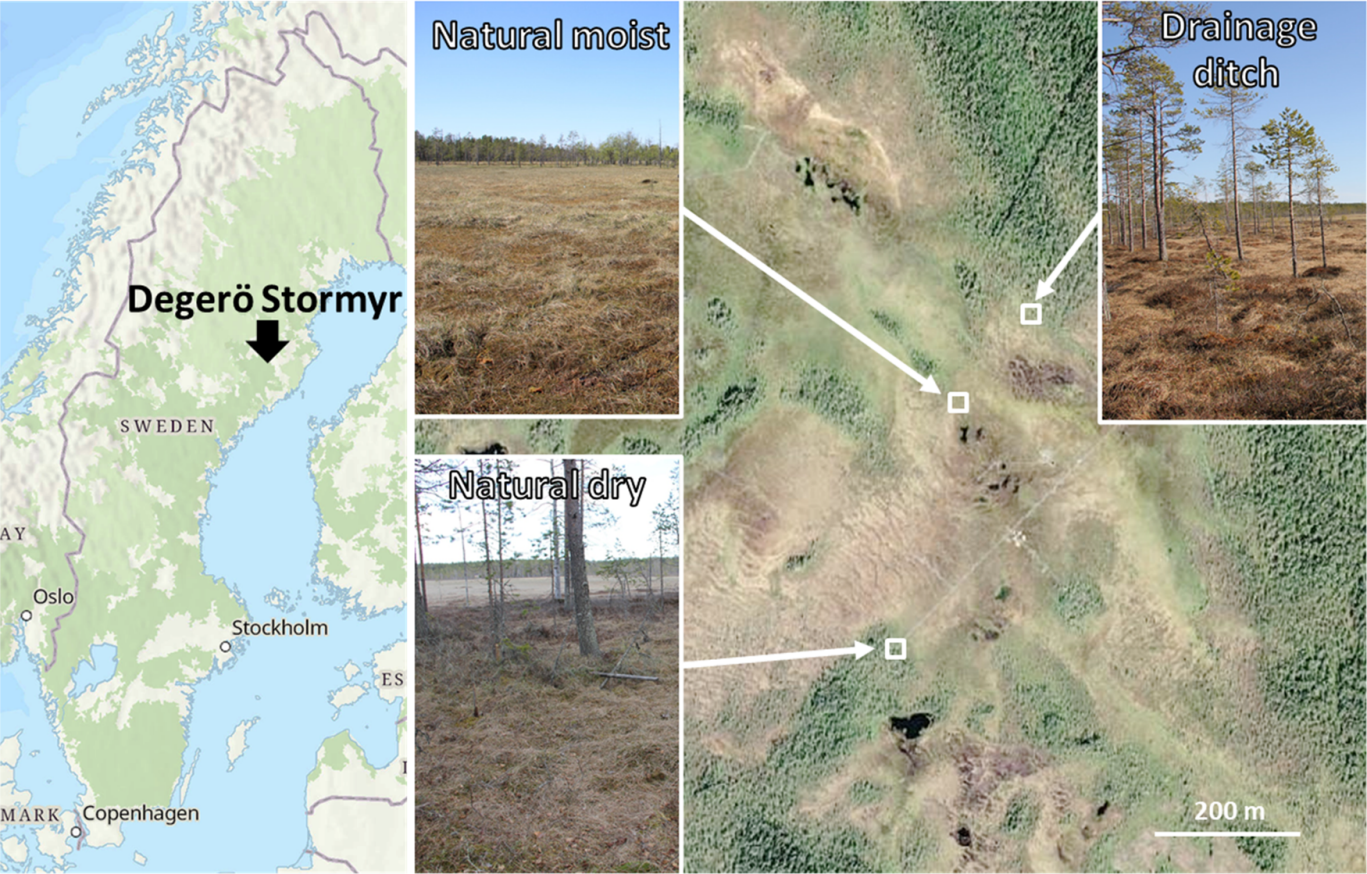
Location of the Degerö-Stormyr and the three sampling sites: natural moist, drainage ditch, and natural dry. Map and satellite image are from ArcGIS Online, Esri USGS (©October 25, 2022).

Peat cores were retrieved from three different sites at the Degerö-Stormyr, including the center of an assumingly natural site with moist conditions (NM, 64°11.042, 19°33.386), a natural dry site (ND, 64°10.812, 19°33.166), and a site in 1 m distance to a drainage ditch (DD, 64°11.090, 19°33.469; Figure 1). For each site, three peat cores were extracted with a Russian peat corer (Eijkelkamp, The Netherlands). The cores were placed in plastic shells, wrapped with plastic foil, stored in coolers, and transported to the laboratory. The cores were sliced into 2 cm sections, and every other section, between 0 and 40 cm, was used for subsequent chemical analyses yielding a total of 90 samples. Samples were oven-dried at 40 °C for 72 h before further analysis.

### 2.2 Site Description

Degerö-Stormyr is a nutrient-poor minerogenic peatland in northern Sweden (Figure 1, 64°11′N, 19°33′E, 270 m asl) near Vindeln municipality and is part of the ICOS (Integrated Carbon Observation System)^43^ Swedish national and European research infrastructure. The peatland has a size of about 6.5 km^2^ and consists of interconnected smaller mires separated by islets and ridges.^7^ The climate is defined as cold temperature humid, with a mean annual air temperature of 1.2 °C and mean annual precipitation of 523 mm.^44^ The peat depth is between 3 and 4 m. Drainage ditches were constructed at the beginning of the 20th century.^7^ At the natural moist site (NM), the water table is near the surface; at the drainage ditch (DD), it is 10–15 cm below the surface; and at the natural dry (ND) site, it is 30–40 cm below surface. The vegetation of the NM site is dominated by *Sphagnum majus* Russ. C. Jens, the DD site by *Sphagnum balticum* Russ. C. Jens, and the ND site by *Sphagnum fuscum*. The vascular plant community at all sites, includes mostly *Eriophorum vaginatum* L., *Trichophorum cespitosum* L. Hartm., *Vaccinium oxycoccos* L., *Andromeda polifolia* L., and *Rubus chamaemorus* L.^7^

### 2.3 2D NMR Spectroscopy

The samples were prepared and analyzed by 2D NMR according to Soucémarianadin et al.^24^ with slight modifications. Briefly, between 100 and 500 mg peat was ground in a 50 mL ZrO_2_ jar with 10 mm ZrO_2_ balls using a planetary ball mill (Fritsch Pulverisette 7, Germany), for 1 h 50 min using 20 min milling intervals with 10 min breaks in between to avoid excessive heating of the sample. An aliquot of the sample (10 mg) was transferred to a standard NMR tube and dissolved in DMSO-*d*_6_ (600 μL, Sigma-Aldrich) by stirring, vortex-mixing for 30 s, sonicating for 10 min, and again vortex-mixing for 30 s. The samples were left to settle for about 48 h to avoid precipitation effects from excess material. The sample did not dissolve completely, and a small pellet was formed. Previous results showed no significant difference in composition between the original sample and the dissolved fraction.^24^ The 2D NMR spectra were acquired with a sensitivity improved ^1^H–^13^C heteronuclear single quantum coherence (HSQC) pulse sequence (hsqcetgpsisp2.2) using a 600 MHz Bruker AVANCE III spectrometer (Bruker Biospin), equipped with a triple resonance cryo-probe. Smoothed square gradients were used for coherence selection. Sweep widths of 8.1 ppm and 140 ppm were used in the ^1^H and ^13^C dimensions, respectively. Each experiment consisted of 16 scans for each of the 300 t_1_ increments. The relaxation delay was 2 s.

### 2.4 Spectral Processing and Analyses

**Figure 2:**
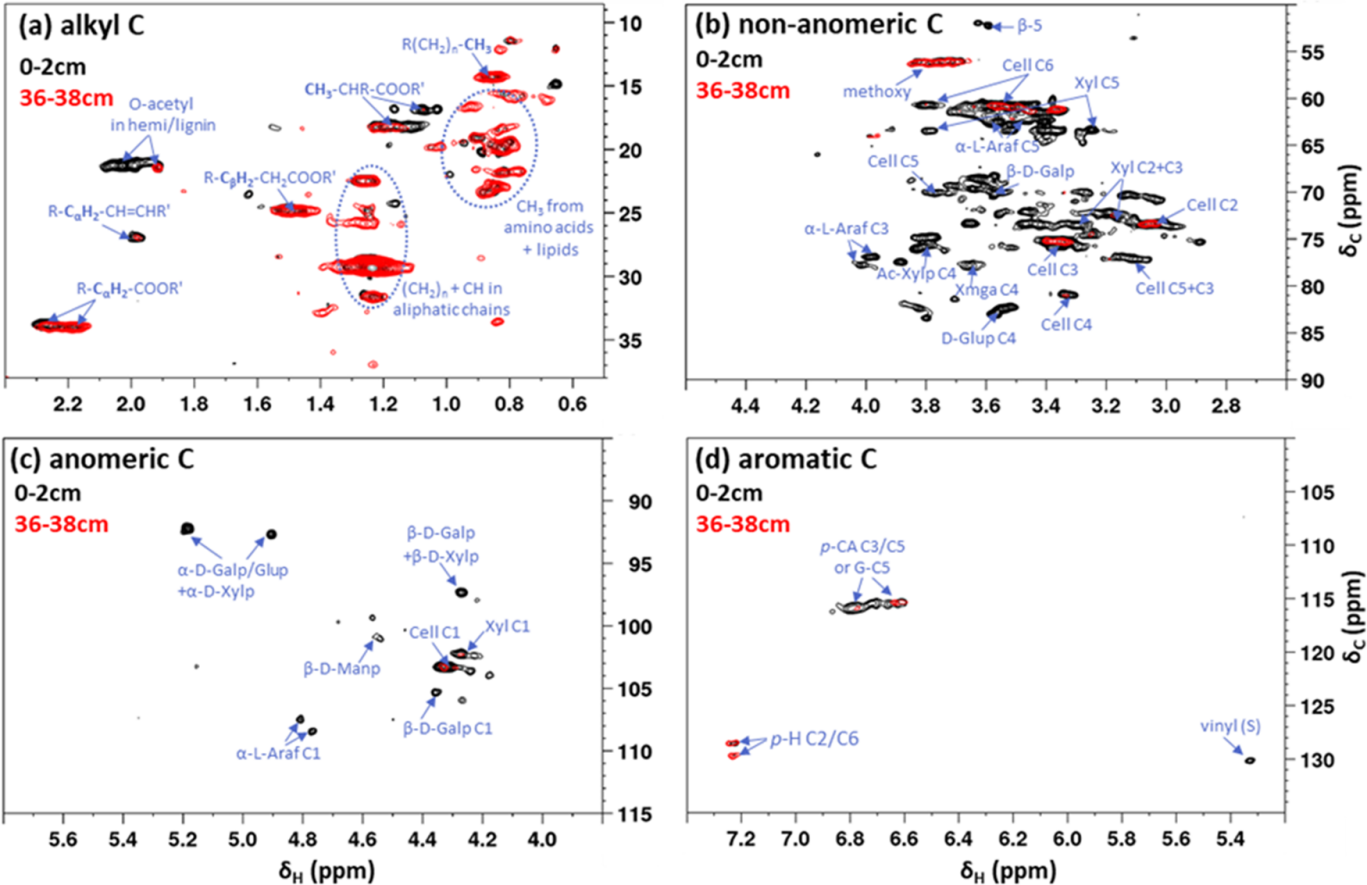
Comparison of the alkyl (a), nonanomeric (b), anomeric (c), and aromatic (d) C region between young peat (0–2 cm, black) and older peat (36–38 cm, red) from one peat core of the natural dry site by overlaying ^1^H–^13^C HSQC NMR spectra. Signal assignments of major peaks are indicated in each caption. Cell, cellulose; Xyl, xylan; Araf, arabinofuranose; Galp, galactopyranose; Glup, glucopyranose; Xylp, xylopyranose; Manp, mannopyranose; Xmga, 4-*O*-methyl-α-d-glucuronic acid. See Table S1 for other abbreviations.

Spectral processing was performed using TopSpin software (Version 3.6.3, Bruker Biospin). A squared sine-bell window function was applied in both dimensions using a sine-bell shift of 2.2 and 2.4 in the ^1^H and ^13^C dimensions, respectively. In the ^13^C dimension, the number of data points was extended by linear prediction (prediction to 512 points using 40 coefficients). Baseline correction was performed using a fifth-order polynomial, and all spectra were manually phase-corrected and calibrated before analysis. Automatic peak picking was performed on selected regions: (a) the alkyl C region (^1^H chemical shift (δ_H_): 0.5–2.5 ppm, ^13^C chemical shift (δ_C_): 5–40 ppm), (b) the nonanomeric C region (δ_H_: 2.5–6 ppm, δ_C_: 45–90 ppm), (c) the anomeric regions (δ_H_: 4–5.5 ppm, δ_C_: 90–110 ppm), and (d) the aromatic C region (δ_H_: 3.5–8 ppm, δ_C_: 90–140 ppm; Figure 2 and Table S1). The anomeric C represents the aldehyde or keto group of a sugar, in contrast to the nonanomeric CHOH groups in a sugar molecule. Solvent cross-peaks were avoided when selecting these regions. Artifacts selected by automatic peak picking were manually removed prior to peak integration, resulting in a total of 117 integrals. Relative abundances were calculated by dividing the integral of each peak by the sum of all integrals, from all signals detected. The integrals/signals were assigned to specific molecular moieties by comparing their chemical shifts with literature data (Table S1). Out of the 117 signals, 88 signals were assigned, which accounts for ∼91% of the total peak volume (DMSO peak excluded).

### 2.5 Measurements of C, δ^13^C, N, and δ^15^N

Stable C and N isotopic compositions and the C and N content were measured with an elemental analyzer combined with an isotope ratio mass spectrometer (EA-IRMS; Inegra2, Sercon Limited, Crewe, U.K.). Carbon isotopic composition (^13^C/^12^C) was expressed relative to the Vienna Pee Dee Belemnite (VPDB) standard and reported in delta notation (‰). Stable nitrogen isotopic composition (^15^N/^14^N) was expressed relative to the atmospheric nitrogen standard and reported in delta notation (‰). The C/N ratio was determined by the mass ratio of the measured total C and N content.

### 2.6 Statistical Analysis

The principal component analysis (PCA) model of the 117 NMR signals generated three significant principal components (PC1, PC2, and PC3), with explained variances (*R*^2^X) of 26.1, 18.0, and 5.9%, respectively. Adding additional components did not improve the model significantly, explaining less than 4.2% each. To test if the differences between sites and/or depth are significant, we performed partial least squares–discriminant analysis (PLS-DA; Figure S1). The PLS-DA model consists of three predictive components with explained variances in the *X*- and *Y*-matrices (*R*^2^*X* and *R*^2^*Y*) of 24.6 and 26.8 for PC1, 8.5 and 24.8 for PC2, and 13.2 and 10.1 for PC3, respectively. The statistical significance was *p* = 2.798 × 10^–28^, indicating that the model is highly significant. In addition, we performed PLS analysis to test for correlation between the NMR signals (*X* variables, *J* = 117) and the C and N isotopic and elemental composition (*Y* variables; Figure S2). The model generated three components (PC1, PC2, and PC3), which explained variances of 26, 17.5, and 5.7%, respectively, with additional components having minor effects (<4.3% each). The clusters in the PCA and PLS (Figure S2) were defined according to the presence of scores/loadings in respective quadrants. *Q*^2^ represents the predicted fraction according to cross-validation of the variation of the *X* variables in the PCA or of the *X* and *Y* variables in the PLS and PLS-DA, and was 0.42, 0.73, and 0.52 for each model, respectively. The models were generated using the SIMCA-P software package version 16 (Umetrics, Umeå, Sweden). All other statistical analyses were performed in Excel.

**Figure 3:**
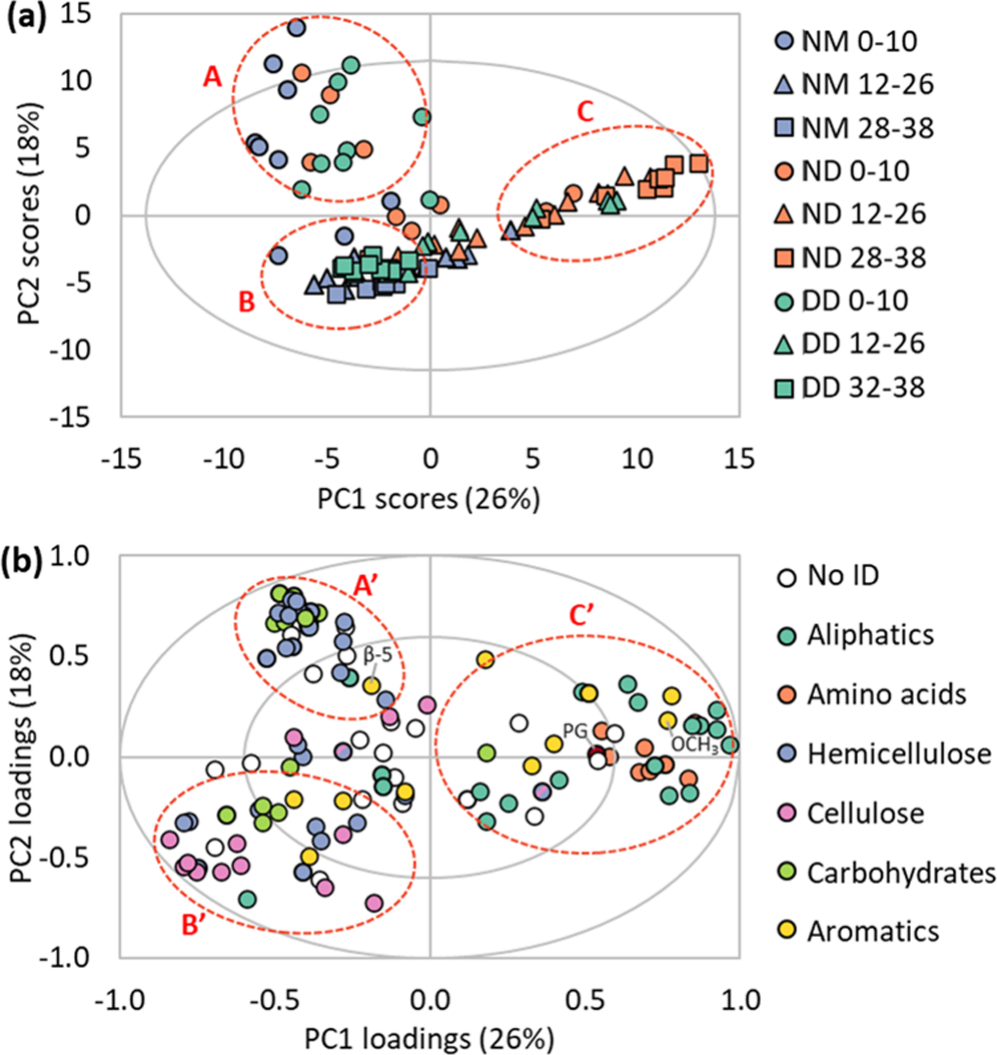
PCA scores (a) and loadings plot (b) with *R*_2_*X*: 0.50, *Q*^2^: 0.42. Model consists of three predictive components (PC1 = 26%, PC2 = 18%, PC3 = 6%). Abbreviations: NM, natural moist site; DD, drainage ditch; ND, natural dry site. No ID, unknown signal origin; amino acids: methyl groups of amino acids and lipids; carbohydrates: other than cellulose and hemicellulose; aromatics: from lignin, cutin, suberin, and tannin. Ellipse in (a) indicates confidence limit of 95%. Ellipses in (b) correspond to correlation scales of 0.6 and 1.0. Red circles in (a) and (b) indicate defined correlation clusters. PG, N-acetyl-group from peptidoglycan; OCH_3_, methoxy-group from lignin.

## 3 Results

### 3.1 Characterization of Peat by 2D NMR

The most abundant signals of the alkyl region were derived from CH_2_ and CH groups ((CH_2_)*_n_* + CH) in aliphatic chains and CH_2_ and CH_3_ groups adjacent to carboxylic acids or esters (R–C_β_H_2_–CH_2_COOR′ and CH_3_–CHRCOOR′) of lipids, fatty acids, and waxes (Figure 2a). At the peat surface (0–2 cm), these signals accounted on average for 15.9 ± 2.8% (SD) of the total integrated peak area of all signals detected, while at 36–38 cm depth, the abundance was 31.0 ± 14.2% (SD) on average. The biggest contribution to the anomeric and nonanomeric C region comes from signals of cellulose subunits (Cell C1–C6, Figure 2b,c), with an average abundance of 27.5 ± 6.4 and 31.4 ± 15.6% (SD) of the total peak area, at 0–2 and 36–38 cm, respectively. Another large contribution comes from hemicellulose-related signals (i.e., xylan and arabinofuranose units), with an average abundance of 17.4 ± 3.1 and 7.3 ± 4.2% (SD) at 0–2 and 36–38 cm, respectively (Xyl C1–C5 and Araf C3–C5, Figure 2b,c). The aromatic region did not contain many signals; most abundant were signals from *p*-coumaryl (*p*-CA C3/C5) and/or guaiacyl (G-C5) units derived from lignin or other (poly-)phenolic compounds with an average of 3.0 ± 0.6 and 3.2 ± 1.1% (SD) at 0–2 and 36–38 cm, respectively (Figure 2d).

### 3.2 Changes in 2D NMR Signals with Depth

To analyze changes in molecular substructures during peat decomposition processes in the acrotelm (the top 40 cm), we initially performed PCA of all signals detected by 2D NMR. PC1 showed a separation of 28–38 cm deep samples from 0 to 10 cm deep samples of the natural dry site (cluster C vs clusters A and B; Figure 3a). In addition, 12–26 cm deep samples from the drainage site clustered with the 28–38 cm deep samples from the natural dry site (cluster C). PC2 showed a separation of surface peat (0–10 cm) from deeper peat (12–38 cm) for samples from the moist and drainage site (cluster A vs cluster B, Figure 3a). PLS-DA confirmed that the separation was highly significant (*p* = 2.798 × 10^–28^; Figure S1). The loadings plot revealed that the separation of surface and older peat (PC2, cluster A vs cluster B) was mostly explained by a higher abundance of cellulose-related signals (89% of all cellulose signals) and a depletion in specific hemicellulose linkages and noncellulosic carbohydrates (e.g., starch) in older peat. The separation of the deeper natural dry site samples from all other samples was related to a higher abundance of nearly all aliphatic (81%) and all amino-acid-related signals with increasing depth (Figure 3b).

**Figure 4:**
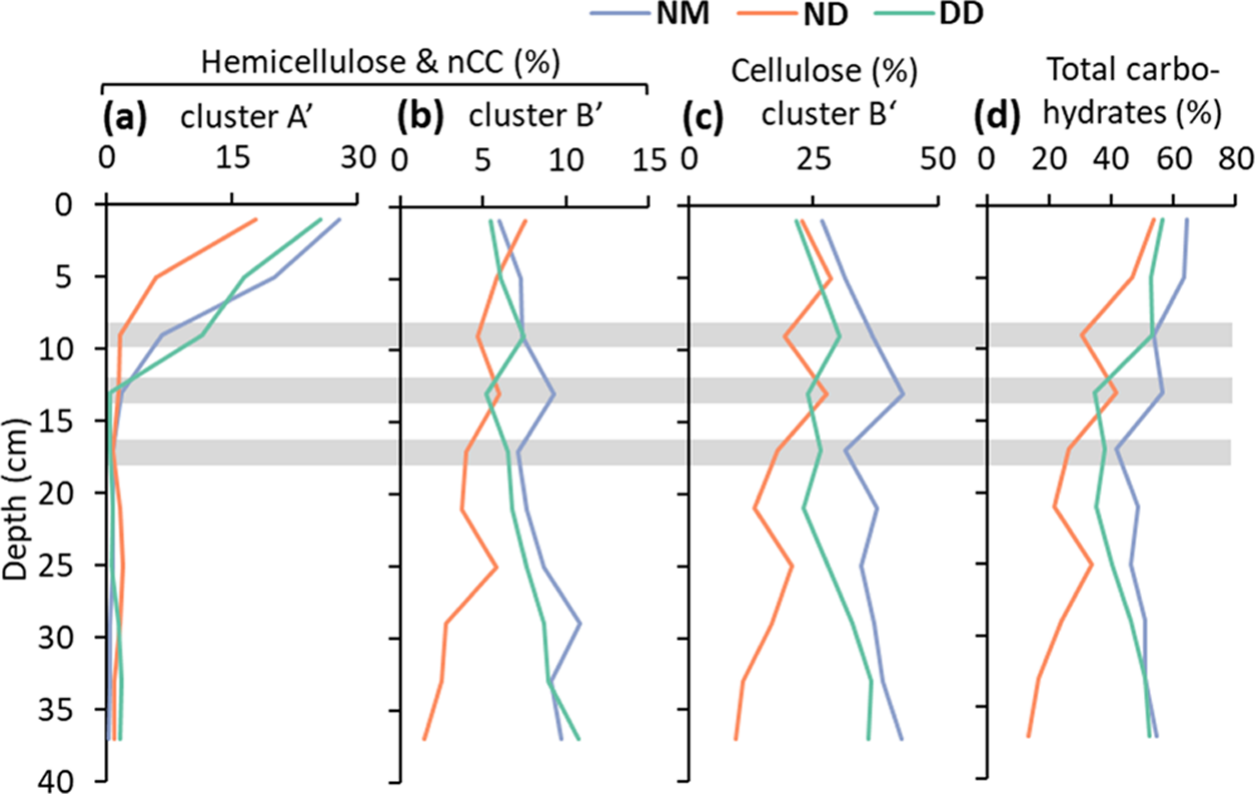
Relative abundance in percent of total integrated peak area of compound-specific 2D NMR signals, grouped according to the presence in PCA-derived clusters A′, B′, and C′ in Figure 3b. Data are the average of three peat cores of the natural moist site (NM, blue), natural dry site (ND, red), and the drainage ditch site (DD, green), respectively. (a) Hemicellulose and noncellulosic carbohydrate (nCC) signals of cluster A′, (b) hemicellulose and nCC signals of cluster B′, (c) cellulose-related signals of cluster B′, and (d) total carbohydrates. Gray horizontal lines are for comparison of site-specific changes and indicate depths of 8–10, 12–14, and 16–18 cm depth, respectively.

To evaluate specific changes in the structural composition of vertical depth profiles, we regressed the total abundance of the hemicellulose linkages and noncellulosic carbohydrate signals from cluster A′ with depth. At all sites, these signals were strongly depleted between 0 and 14 cm depth. The natural moist and drainage ditch both showed the strongest depletion with 26.0 ± 4.5% (average ± SE) and 25.0 ± 1.7%, respectively, between 0 and 14 cm (*p* = 0.014 and 0.001, respectively). The natural dry site showed a depletion of 16.2 ± 3.6% between 0 and 10 cm (*p* = 0.018; Figure 4a). Most of these signals derived from xylan end units (C1, C2, C3), arabinofuranose units (C1, C3, C5), 4-*O*-methyl-α-d-glucuronic acid units (C4), 3-*O*-acetylated xylopyranose (C4), and *O*-acetyl groups of hemicellulose. These signals accounted on average for 70 ± 8% SD of all hemicellulose signals in surface peat. Cluster A′ also contained signals from starch (C1–C4) and phenylcoumaran linkages (β-5) of lignin, which all decreased with depth (Figure S3 and Table S2).

Several hemicellulose and noncellulosic carbohydrate signals were found in cluster B′ (Figure 3b and Table S2). These signals were derived from internal (mid-chain) xylan units (C position 1–4), and galacto- and mannopyranoses (C1 and C2) potentially from galactomannan-type hemicelluloses.^45^ Their total abundance increased at the moist and drainage site, from 5.7 ± 0.3% (average ± SE) to 10.3 ± 0.5% (*p* = 0.016) but decreased from 7.6 ± 0.3 to 1.5 ± 0.8% (*p* = 0.005) at the natural dry site between 0 and 38 cm depth (Figure 4b). Cluster B′ also contained most cellulose signals (C1–C6, Table S2), which followed the same pattern as the hemicellulose and noncellulosic carbohydrate signals of cluster B′ (Figure 4c). At the natural moist site, the relative contribution of the cellulose signals increased between 0 and 14 cm depth, from 26.7 ± 6.1 to 43.0 ± 2.6% (*p* = 0.050); no significant changes were detected below this depth. At the drainage ditch, the signals did not show major changes down to 22 cm but increased from 24.7 ± 3.6 to 36.3 ± 0.7% between 22 and 38 cm (*p* = 0.023). In contrast, the natural dry site showed a gradual decrease in cellulose between 0 and 38 cm from 22.6 ± 1.7 to 9.3 ± 1.2% (*p* = 0.002, Figure 4c).

Overall, the hemicellulose and noncellulosic carbohydrate signals from cluster A′ accounted on average for 40 ± 6% SD of all carbohydrates in near-surface peat. Total carbohydrates, calculated as the sum of all carbohydrate signals detected, decreased only by 8 ± 1% between 0 and 14 cm, at the natural moist site (*p* = 0.038, Figure 4d). At the drainage ditch and the natural dry site, total carbohydrates tended to decrease between 0–14 and 0–10 cm, respectively. However, the decrease was not significant (*p* = 0.064 and 0.051, respectively). Altogether, this indicates that, particularly at the moist site, the decrease of specific hemicellulose structures of cluster A′ is not reflected in the total carbohydrate content due to concomitant increases in other carbohydrates, mostly cellulose, internal xylan units and galactomannan-type hemicelluloses from cluster B′ (Figure 4b,c).

The aliphatics and methyl groups of amino acids and lipids in cluster C′ increased gradually from 23.1 ± 0.9 to 73.7 ± 2.6% between 0 and 38 cm at the natural dry site (*p* < 0.001; Figure S3). At the natural moist site, they only increased between 0 and 18 cm, from 14.8 ± 3.0 to 35.4 ± 6.9% (*p* = 0.040) and remained stable below this depth. At the drainage ditch, there were no significant changes. Cluster C′ also exhibited a signal from methoxy groups of lignin (OCH_3_), and a microbial marker (N-acetyl groups) potentially derived from peptidoglycans of bacteria (PG, Figure 3b).^46^ Both signals specifically increased with depth at the natural dry site (Figure S3). Signals from the aromatic C region did not show any specific trend (Figure S3).

### 3.3 Relationship between 2D NMR Signals and δ^13^C, δ^15^N, C and N Content

Isotopic signatures (δ^13^C and δ^15^N), C and N contents, and C/N ratios have been proposed as indicators for peat decomposition.^28,47,48^ To mechanistically connect the changes in these indices with 2D NMR-derived molecular changes, we used PLS analysis. The PLS showed that the separation of the deeper samples of the natural dry site from all other samples (cluster C vs clusters A and B, Figure S2) was mostly explained by changes in the C and N content, which explained 89 and 83% respectively (*R*_PC1_^2^ = 0.89 and 0.83). The C/N ratio and δ^15^N explained 65 and 57% of this variation while δ^13^C only explained 11% (*R*_PC1_^2^ = 0.65, 0.57, 0.11). The separation of the surface peat from deeper peat of the natural moist and the drainage site (cluster A vs cluster B, Figure S2) was also mostly explained by the C and N content, explaining 90 and 89% respectively, whereas the C/N ratio, δ^15^N, and δ^13^C explained 66, 63, and 45% of variance, respectively (*R*_PC2_^2^ = 0.9, 0.89, 0.66, 0.63, and 0.45, respectively).

**Figure 5:**
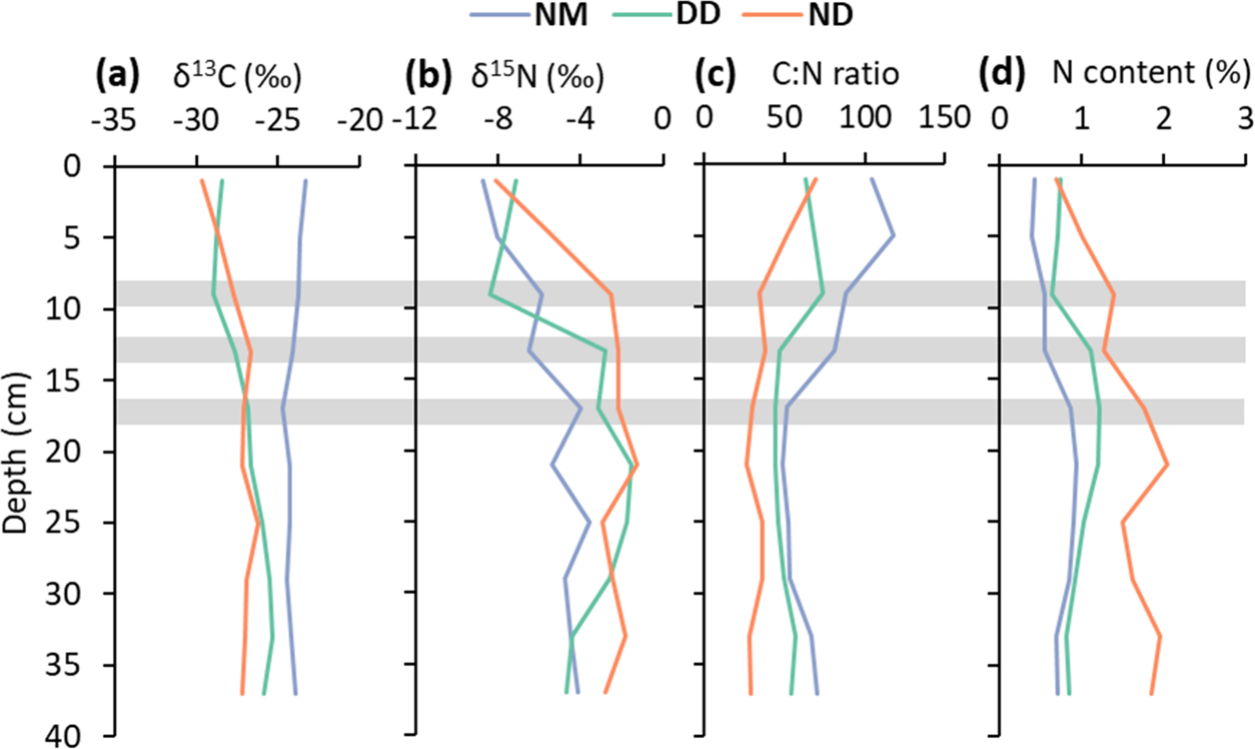
Depth profiles of δ^13^C (a), δ^15^N (b), C/N ratio (c), and N content (d). Data are the average of three peat cores of the natural moist site (NM, blue), natural dry site (ND, red), and drainage ditch site (DD, green) respectively. Gray horizontal lines are for comparison of site-specific changes and indicate depths of 8–10, 12–14, and 16–18 cm depth. respectively.

The results of the PLS model suggested specific relationships between δ^13^C, δ^15^N, C and N content, and the molecular composition. Therefore, we compared stratigraphic changes in these parameters (Figure 5) with the relative abundance of compound-specific NMR signals from clusters A′, B′, and C′ (Figure 4). The three sites showed different patterns in isotopic and elemental composition. δ^13^C did not show any major changes except that the natural moist site had higher values (higher ^13^C content) than the natural dry and drainage sites (Figure 5a). δ^15^N slowly increased between 0 and 40 cm depth at the natural moist site. In contrast, the natural dry site showed strong increases in δ^15^N (from −8.1 to −2.5‰) between 0 and 10 cm (Figure 5b). The drainage ditch also showed a strong increase in δ^15^N (from −8.4 to −2.8‰) but between 8 and 14 cm. The C/N ratio decreased by 35 units between 0 and 10 cm at the natural dry site, and by 27 units between 10 and 15 cm at the drainage ditch (Figure 5c). At the natural moist site, the C/N ratio strongly decreased between 4 and 18 cm depth, by 60 units. The N content did not show major changes, except for the natural dry site where it increased from 0.7 to 2% between 0 and 22 cm (Figure 5d).

## 4 Discussion

### 4.1 Molecular Changes during Peat Decomposition

**Figure 6:**
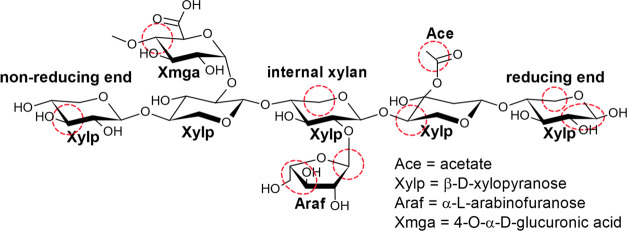
Schematic presentation of preferentially degraded C-linkages (red circles) of xylan-type hemicelluloses.

Our data indicate site-specific differences in the molecular composition of peat. When comparing surface and older peat of the natural moist and drainage sites, most cellulose signals tended to increase with depth, indicating that cellulose accumulates at these sites (Figure 4c). In contrast, at the dry site, cellulose was nearly completely degraded. This supports the findings by Schellekens et al.,^21^ showing that cellulose is only weakly degraded under anaerobic/water-saturating conditions. Regarding hemicellulose, we found differences depending on molecular moieties. Most hemicellulose signals from internal units showed a pattern like cellulose, whereas signals from side branches and end units highlighted in Figure 6, decreased sharply in the upper 14 cm for all sites. This indicates that hemicellulose side branches and end units are preferentially degraded at all sites. In contrast, the backbone remains relatively intact at the moist and drainage site but was nearly completely degraded at the natural dry site.

In contrast to cellulose, starch was quickly degraded in the upper 14 cm (Cluster A′, Figures 3b and 4a), which agrees with their difference in structure, i.e., the straight chains of cellulose allow crystallization in contrast to starch. Concerning lignin, we found that β-5 linkages decrease quickly in the upper 14 cm of the drainage and natural dry site (Figure S3), indicating structural changes of lignin.^49,50^ The methoxy groups of lignin units did not change significantly at the natural moist and drainage site but increased with depth at the natural dry site (Figure S3). This suggests that at the natural dry site, lignin is structurally modified in the upper 14 cm and subsequently accumulated in deeper peat.^50,51^ Vegetational changes may affect lignin content and composition as well.^21^ However, the abundance of lignin-related compounds detected was low, suggesting that vascular plant cover was low during history at all sites.

The hemicellulose side branches, accounting for 70% of all hemicellulose signals, were degraded rapidly within the upper 14 cm at all sites, whereas cellulose degraded slowly below 15 cm and specifically at the natural dry site (Figure 4). In plant cell walls, cellulose is present in the form of crystalline microfibrils coated with hemicellulose and pectin, which hinders degradation due to inaccessibility.^52^ This could explain the successive decay of hemicellulose first and then cellulose at the natural dry site. This mechanism also suggests that different microorganisms are involved in their degradation. In the acrotelm (0–20 cm), fungal microbes are most abundant as fungi are better adapted to low moisture conditions, whereas in the mesotelm (20–50 cm), bacterial communities become more abundant.^30,53,54^ Thus, aerobic fungi are probably involved in the decomposition of specific hemicellulose linkages in the upper 14 cm. The bacterial marker (N-acetyl of peptidoglycan) increased at the natural dry site below 10 cm (Figure S3), which also agrees with an increase in bacterial abundance with depth at the dry site.

### 4.2 Ecophysiological Implications of Peat Molecular Changes

Using 2D ^1^H–^13^C NMR, we followed molecular changes in the acrotelm and upper mesotelm (upper 40 cm). Previous studies often used the *O*-alkyl C region of ^13^C NMR (60–110 ppm) as an indicator of carbohydrate decomposition.^16,55^ Our analysis revealed that this region contains various types of carbohydrate linkages including hemicellulose, cellulose, and starch. In addition, we found that 11 ± 6% of the *O*-alkyl C region consists of fragments of lignin- and aliphatic-related compounds (Table S1). These compounds generally accumulate with depth, which indicates that carbohydrate decomposition rates estimated by conventional ^13^C NMR may be underestimated.

Quantification of the NMR signals revealed that at all sites specific linkages of hemicelluloses and other noncellulosic carbohydrates (mostly starch) were degraded in the acrotelm (within the upper 14 cm). The relative abundance of these signals at the surface (0–2 cm) of the natural moist and drainage sites was ca. 25% of all signals detected and 16% at the natural dry site. This indicates that up to one-fourth of the recently deposited peat litter is lost in the acrotelm. These labile hemicellulose linkages account for 70% of all hemicellulose signals detected. In addition, they mostly derived from xylan-type hemicelluloses, whereas signals from galactomannan-type hemicelluloses rather increased with depth (cluster B′, Figure 4b). This indicates that hemicellulose content and composition play an essential role in the stability of surface peat. Increased temperatures and latitudinal gradients were found to influence the carbohydrate content of peat,^56−58^ indicating that differences in specific carbohydrate linkages are important for peat stability. Moreover, it was found that CO_2_ releases were twice as high in surface peat (2.5–5 cm) than in older peat (10–12.5 cm).^59^ Altogether, this suggests that decomposition of labile hemicellulose linkages in the acrotelm substantially contributes to C emissions and thus plays an important role in peat stability in response to climate change.

The hemicellulose content varies greatly between different *Sphagnum* taxonomic sections,^60^ suggesting that decomposition processes in the acrotelm depend on the type of species. In our study, the dry site had significantly lower cluster A′ hemicellulose signals between 0 and 14 cm, compared to the natural moist and drainage sites (Figure 4a). The dry site is dominated by species of the section Acutifolia, compared to the drainage ditch and the natural wet site, which are dominated by species of the section Cuspidata. At all sites, the cluster A′ signals decreased simultaneously, indicating site-independent decomposition of these linkages. This is in line with Biester et al.^19^ who found that decomposition processes dominate compared to vegetational differences. However, an initially lower content of specific hemicellulose linkages at the dry site caused these linkages to be already depleted at 10 cm, compared to 14 cm at the other sites. This shift could affect subsequent decomposition processes and microbial metabolism.^30^

### 4.3 Relationship between Molecular Changes and δ^15^N, δ^13^C, and C and N Content

No clear correlation was observed between C/N ratio, C and N content, and specific molecular moieties. The strong decrease in labile hemicellulose linkages was not correlated with the elemental composition. This is most likely related to relative increases in cellulose, which compensates for the overall decrease in hemicellulose side branches. The C content correlated with relative changes in total carbohydrates and aliphatics as the result of changes in the H/C and O/C ratio of the overall peat composition.^14,42^ The N content is also highly correlated with the aliphatics (Figure S2) potentially because of the relative enrichment in N-containing compounds.^17,42^ In summary, C/N ratio and C and N content do not provide information on specific carbohydrate linkages but rather reflect changes in total carbohydrates and aliphatics.

No strong correlation was observed between δ^13^C, δ^15^N, and molecular composition (Figure S2). The decomposition of specific linkages apparently does not have direct effects on bulk isotopic signatures. The specific degradation of hemicellulose side branches (from cluster A′) did not appear to be directly linked to changes in δ^13^C or δ^15^N. The δ^13^C values appear to be more controlled by the different hydrological conditions at the sites (moist vs dry and drained, Figure 5). Nevertheless, the strong increase in δ^15^N at the natural dry and drainage sites (at 0–10 and 8–14 cm, respectively) was accompanied by an increase in aliphatics and a decrease in total carbohydrates (Figures 5b, 4d, and S3). Increases in δ^15^N have previously been linked to increased microbial abundance and/or changes in microbial communities.^30^ This suggests that the increased decomposition of carbohydrates and relative accumulation of aliphatics is linked to shifts in microbial abundance and/or composition. The shift in δ^15^N toward more positive values might be caused by the release of “lighter” (^14^N-enriched) N from the peat, but high losses of N are unlikely in natural N-limited peatlands.^61^ Therefore, considering the conditions at the investigated sites, the shift in δ^15^N probably results from the uptake of “lighter” N by vascular plants through root-mycorrhizal interactions.^38^

## Supporting Information Available

Figure S1: Scores and loadings plot of the PLS-DA of the NMR signals. Figure S2: PLS scores and loadings plot of the NMR signals, with δ^13^C, δ^15^N, C and N content, C/N ratio, and peat depth as *Y* variables. Figure S3: Relative abundance of aliphatics and methyl groups of amino acids, β-5 linkages of lignin, methoxy groups of lignin, N-acetyl groups of peptidoglycans, *p*-hydroxyphenyl units, and *p*-coumaryl units. Table S1: Assignments of ^1^H and ^13^C chemical shifts to molecular moieties in peat. Table S2: Molecular moieties of clusters A′, B′, and C′ from the PCA in Figure 3 (PDF)

## Notes

Dataset 1: Relative abundance of the NMR signals. Dataset 2: C and N elemental and isotopic composition. The datasets generated during analysis are available through Zenodo.

## Abbreviations

2D NMR: two-dimensional NMR spectroscopy
OM: organic matter
NM: natural mire site
ND: natural dry site
DD: drainage ditch site
PCA: principal component analysis
PLS: partial least-square analysis
