# Supplementary material for: Organochemical Characterization of Peat Reveals Decomposition of Specific Hemicellulose Structures as the Main Cause of Organic Matter Loss in the Acrotelm
Source: Environ Sci Technol. 2022 Nov 18;56(23):17410–9. doi: 10.1021/acs.est.2c03513 (PMC9730845; doi:10.1021/acs.est.2c03513)
Supplement: Supplementary file 1 — es2c03513_si_001.pdf [file es2c03513_si_001.pdf]

SUPPORTING INFORMATION

Organo-chemical characterisation of peat reveals decomposition of specific hemicellulose structures as main cause of organic matter loss in the acrotelm

Henrik Serk[1,2], Mats B. Nilsson[2*], João Figueira[3], Jan Paul Krüger[4,5], Jens Leifeld[5,6], Christine Alewell[5], and Jürgen Schleucher[1*]

[1] Department of Medical Biochemistry and Biophysics, Umeå University, SE-90187 Umeå, Sweden

[2] Department of Forest Ecology and Management, Swedish University of Agricultural Sciences, SE-90183 Umeå, Sweden

[3] Department of Chemistry, Scilife Lab, SE-90187 Umeå University, Umeå Sweden

[4] UDATA GmbH – Umwelt und Bildung, Hindenburgstrasse 1, 67433 Neustadt/Weinstr., Germany

[5] Departement Umweltgeowissenschaften, Universität Basel, Bernoullistrasse 30, CH-4056 Basel, Switzerland

[6] Agroscope, Climate and Agriculture Group, Reckenholzstrasse 191, CH-8046 Zurich, Switzerland

* Corresponding authors: Mats B. Nilsson, e-mail: mats.b.nilsson@slu.se, and Jürgen Schleucher, e-mail: jurgen.schleucher@umu.se

Summary of SI material: 11 total pages, 3 figures and 2 tables.

SUPPLEMENTAL FIGURES

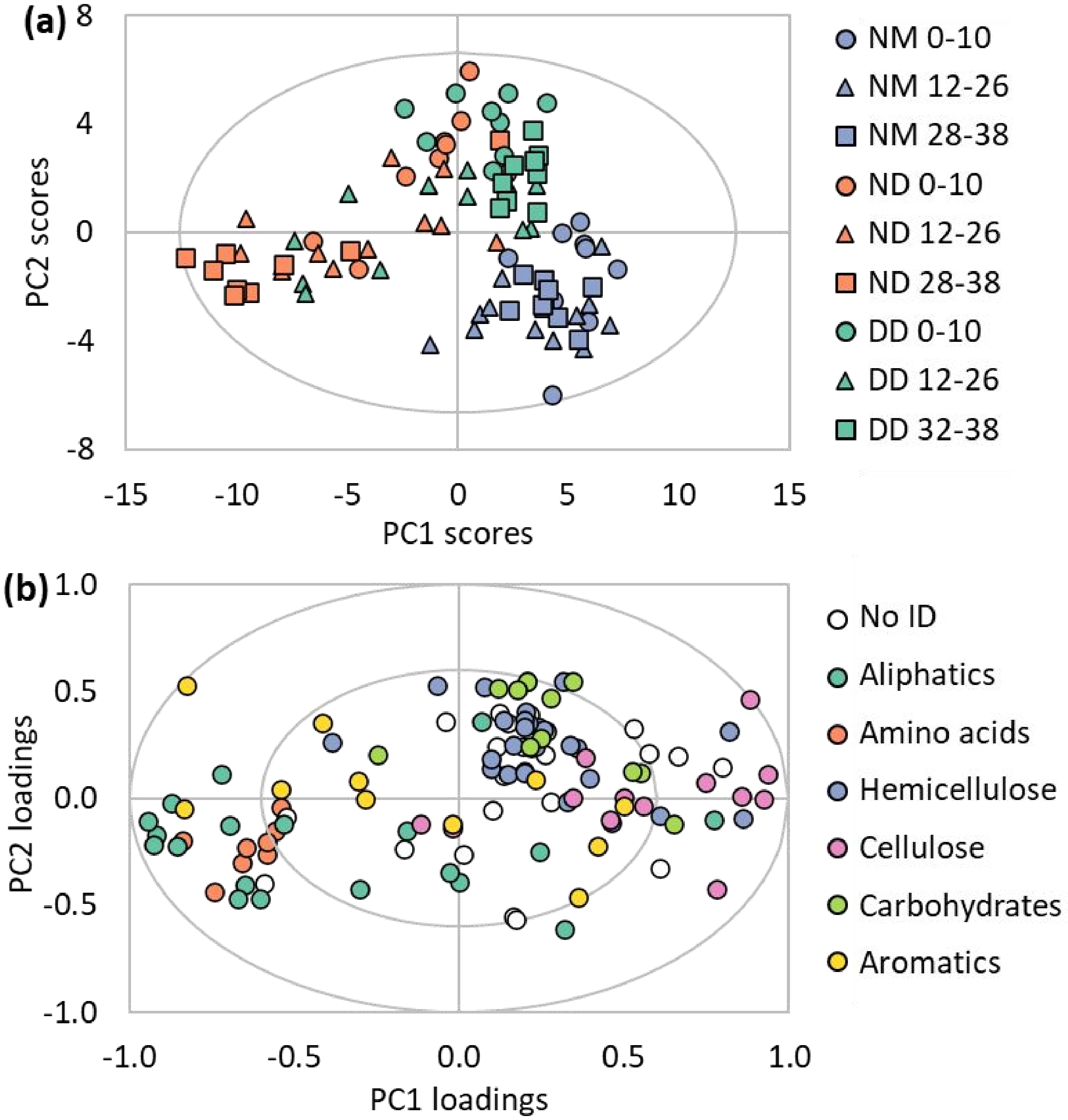

Figure S1. PLS-DA scores (a) and loadings plot (b) with R_2X: 0.46, R_2Y: 0.62, Q_2: 0.52. Model consists of three predictive components with R_2X_1=0.25 and R_2Y_1=0.27 for PC1, R_2X_2=0.09 and R_2Y_2=0.25 for PC2, and R_2X_3=0.13 and R_2Y_3=0.10 for PC3. Statistical significance: P=2.798 e^{-28}. Abbreviations: NM, natural moist site; DD, drainage ditch site; ND, natural dry site. No ID, unknown signal origin; amino acids: methyl-groups of amino acids and lipids; carbohydrates: other than cellulose and hemicellulose; aromatics: from lignin, cutin, suberin and tannin. Ellipse in (a) indicates confidence limit of 95%. Ellipses in (b) correspond to correlation scale of 0.6 and 1.0 respectively.

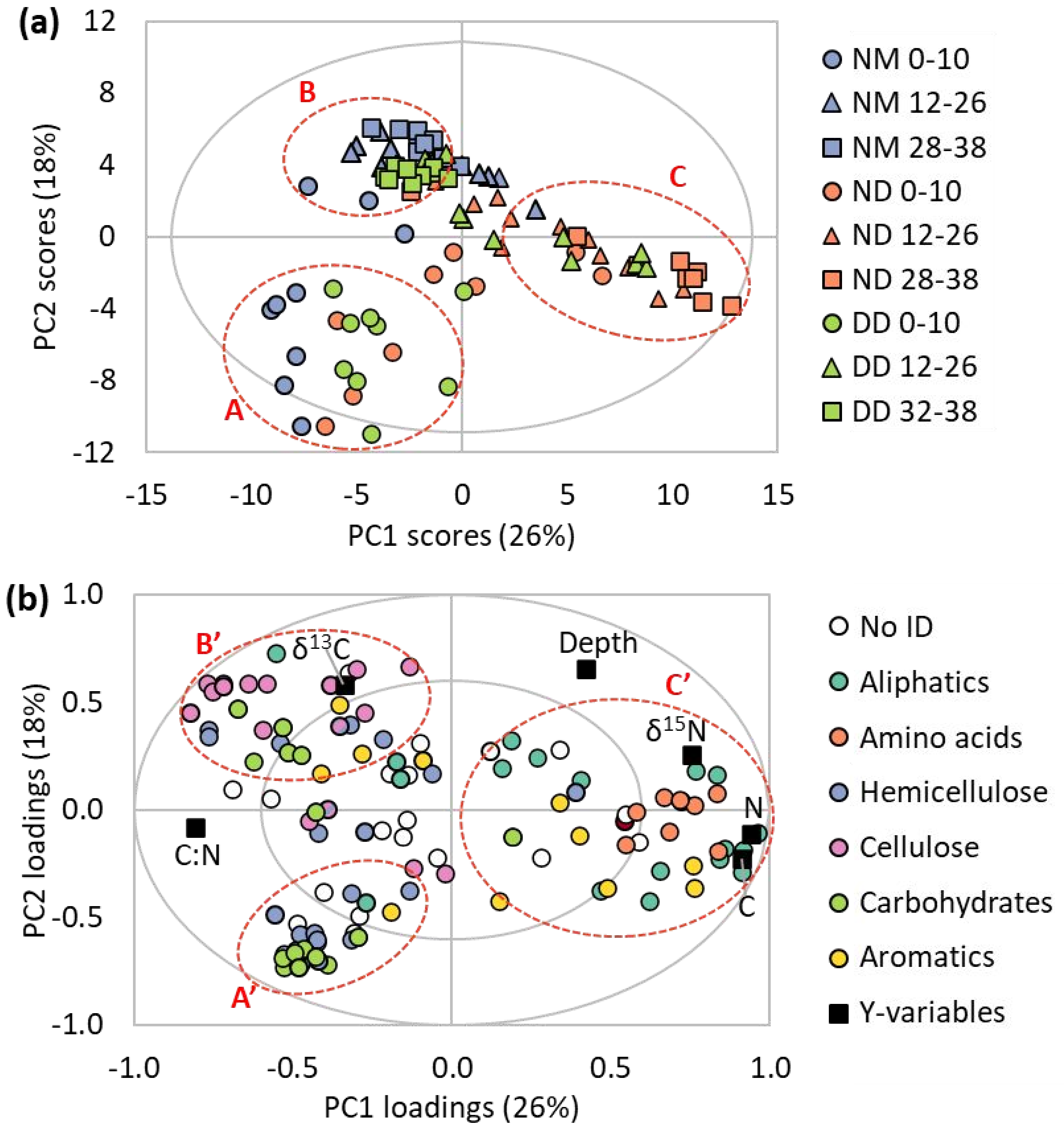

Figure S2. PLS with $\delta^{13}C$, $\delta^{15}N$, C content, N content, C:N ratio and peat depth as Y-variables. PLS scores (a) and loadings plot (b) with R_2X: 0.49, R_2Y: 0.77, Q_2: 0.73. Model consists of three predictive components (PC1=26%, PC2=18%, PC3=6%). Abbreviations: NM, natural moist site; DD, drainage ditch site; ND, natural dry site. No ID, unknown signal origin; amino acids: methyl-groups of amino acids and lipids; carbohydrates: other than cellulose and hemicellulose; aromatics: from lignin, cutin, suberin and tannin. Ellipse in (a) indicates confidence limit of 95%. Ellipses in (b) correspond to correlation scale of 0.6 and 1.0 respectively. Red circles in (a) and (b) indicate defined correlation clusters A, B and C.

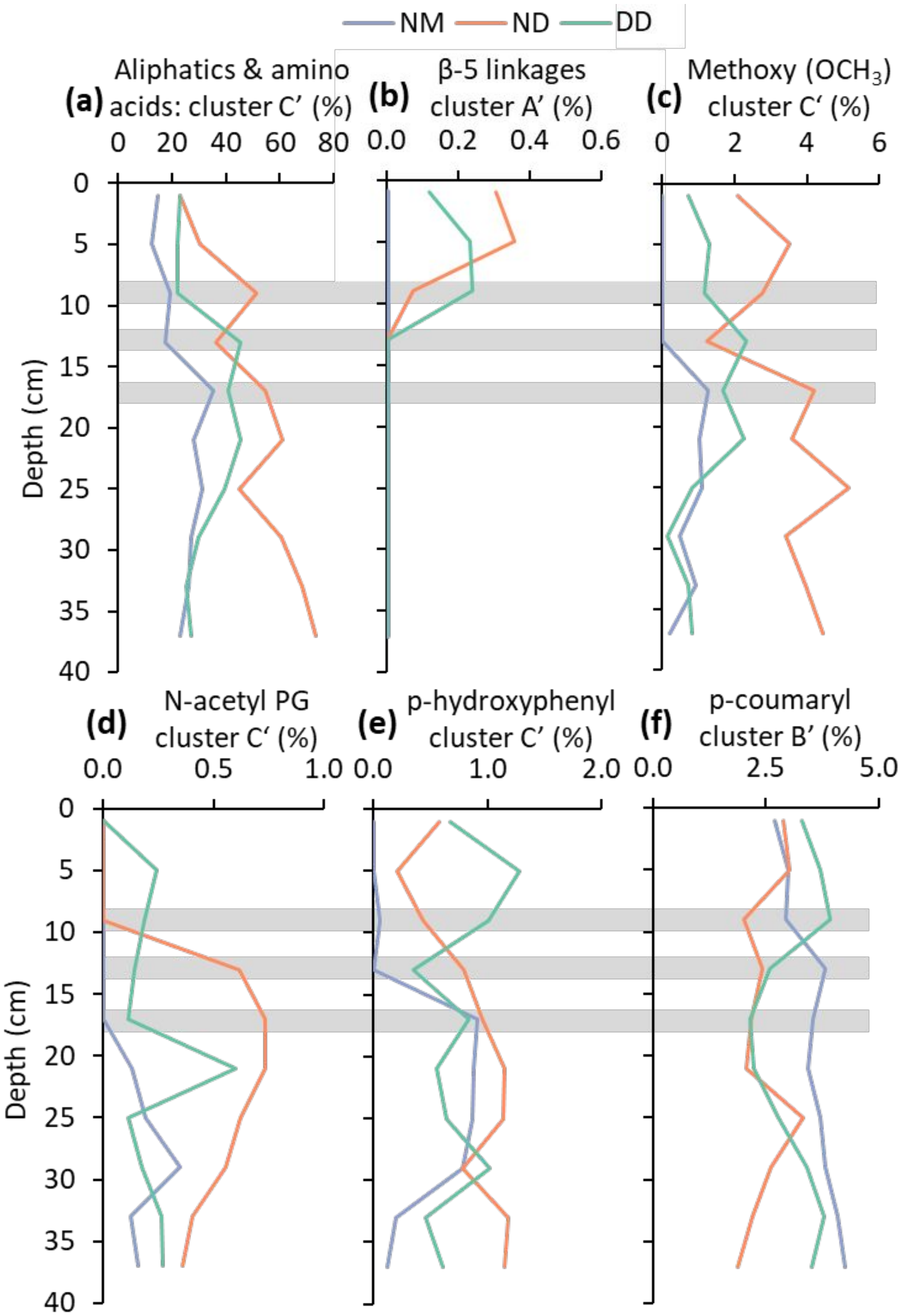

Figure S3. Relative abundance in percent of total integrated peak area of aliphatics and methyl-groups of amino acids (a), β-5 linkages of lignin (b), methoxy-groups of lignin (c), N-acetyl groups of peptidoglycans (d), p-hydroxyphenyl units (e) and p-coumaryl units (f). Data are the average of three peat cores of the natural moist site (NM, blue), natural dry site (ND, red), and the drainage channel site (DD, green). Grey horizontal lines are for comparison of site-specific changes and indicate depth of 8-10, 12-14 and 16-18 cm depth respectively.

SUPPLEMENTAL TABLES

Table S1. Assignments of ^{1}H and ^{13}C chemical shifts to molecular moieties in peat.

Moiety	Abbreviation	δC (ppm)	δH (ppm)	References
Dimethyl sulfoxide (solvent)	DMSO	40	2.50	Soucémarianadin et al., 2017
**Alkyl C region**				
Methylene units in aliphatic chains **α** to carboxyl acid or carbonyl of an ester	R-$\mathbf{C_{\alpha}H_2}$-COOR'	34.1<br>34.0	2.18<br>2.26	Deshmukh et al., 2003;<br>Soucémarianadin et al., 2017
Methylene units in aliphatic chains **β** to carboxyl acid or carbonyl of an ester	R-$\mathbf{C_{\beta}H_2}$-CH_2COOR'	24.9<br>26.7	1.48<br>1.56	Hertkorn et al., 2015;<br>Soucémarianadin et al., 2017
Methylene **β** in a primary alcohol chain in waxes	R-$\mathbf{C_{\beta}H_2}$-CH_2OH	33.6<br>32.9	1.52<br>1.39	Deshmukh et al., 2003<br>Soucémarianadin et al., 2017
Methylene and methine groups in aliphatic chains: polymethylene chains from lipids, fatty acids and waxes	$(CH_2)_n$ + CH	31.7<br>29.4<br>25.9<br>24.8<br>22.5	1.24<br>1.23<br>1.26<br>1.25<br>1.26	Simpson et al., 2007;<br>Soucémarianadin et al., 2017
Methylene units **β** to oxygen of an ester	R-$\mathbf{C_{\beta}H_2}$-CH_2OCOR'	28.7	1.52	Deshmukh et al., 2003
Methylene units **α** adjacent to alkene	R-$\mathbf{C_{\alpha}H_2}$-CH=CHR'	27.0	1.98	Soucémarianadin et al., 2017
N-acetyl groups from peptidoglycan	R-NHCO$\mathbf{CH_3}$	23.4	1.83	Simpson et al., 2007
O-acetyl groups of hemicellulose	R-COO$\mathbf{CH_3}$	21.5<br>21.4	1.91<br>2.02	Kim & Ralph, 2010;<br>Soucémarianadin et al., 2017
Methyl groups adjacent to carboxyl acid or carbonyl/oxygen of an ester	$\mathbf{CH_3}$-CHR-COOR'	18.3<br>17.0	1.13<br>1.09	Hertkorn et al., 2015
Methyl groups from amino acids in peptides/proteins, and branched methyl groups in lipids and fatty acids	$\mathbf{CH_3}$ from amino acids + lipids	23.0<br>22.1<br>21.9<br>20.0<br>19.9<br>19.1<br>18.5<br>16.8<br>15.8<br>12.2	0.84<br>0.99<br>0.82<br>0.82<br>1.03<br>0.90<br>0.82<br>0.92<br>0.80<br>0.82	Kelleher & Simpson, 2006;<br>Simpson et al., 2007;<br>Hertkorn et al., 2002, 2015;<br>Soucémarianadin et al., 2017<br>Sahakyan et al., 2011
Terminal methyl groups of aliphatic chains	R-$(CH_2)_n$-$\mathbf{CH_3}$	14.4	0.85	Soucémarianadin et al., 2017
Methyl groups in waxes	$\mathbf{CH_3}$ from waxes	11.5	0.79	Soucémarianadin et al., 2017
**Non-Anomeric C region**				
C4/H4 of (1→4) β-D-glucopyranose	D-Glup C4	82.5	3.54	Komatsu & Kikuchi, 2013
Starch				
C3/H3 of (1→4)-α-D-glucopyranose (starch)	Starch C3	74.9	3.79	Komatsu & Kikuchi, 2013
C4/H4 of (1→4)-α-D-glucopyranose (starch)	Starch C4	73.4	3.64	Komatsu & Kikuchi, 2013
C5/H5 of (1→4)-α-D-glucopyranose (starch)	Starch C5	73.3	3.45	Komatsu & Kikuchi, 2014
C2/H2 of (1→4)-α-D-glucopyranose (starch)	Starch C2	70.4	3.13	Komatsu & Kikuchi, 2013
Cellulose				
C4/H4 of internal and reducing ends (α+β) of cellulose	Cell C4 (R+i)	80.9	3.33	Kim & Ralph, 2014
C5/H5 and C3/H3 of internal & non-reducing end of cellulose	Cell C5+C3 (NR+i)	76.9	3.14	Soucémarianadin et al., 2017

C3/H3 of internal & reducing end (β) of cellulose	Cell C3 (R+i)	75.2	3.36	Kim & Ralph, 2014
C2/H2 of reducing end (β) of cellulose	Cell C2 (R)	74.7	3.00	Kim & Ralph, 2014
C5/H5 of reducing end (β) of cellulose & C3/H3 of xylan	Cell C5 (R) & Xyl C3 (i)	74.4	3.25	Soucémarianadin et al., 2017
C2/H2 of non-reducing end of cellulose	Cell C2 (NR)	73.7	2.99	Kim & Ralph, 2014
C2/H2 of internal cellulose	Cell C2 (i)	73.4	3.06	Kim & Ralph, 2014
C2/H2 of reducing end (α) of cellulose & xylan	Cell & Xyl C2 (R)	72.2	3.30	Soucémarianadin et al., 2017
C3/H3 of reducing end (α) of cellulose	Cell C3 (R)	70.9	3.69	Kim & Ralph, 2014
C4/H4 of non-reducing end of cellulose	Cell C4 (NR)	70.2	3.27	Soucémarianadin et al., 2017
C5/H5 of reducing end (α) of cellulose	Cell C5 (R)	70.1	3.77	Kim & Ralph, 2014
C6/H6 (α) of non-reducing-end of cellulose	Cell C6 (NRa)	61.5	3.42	Kim & Ralph, 2014
C6/H6 (β) of non-reducing-end of cellulose	Cell C6 (NRb)	61.5	3.69	Kim & Ralph, 2014
C6/H6 (α) of internal and reducing ends (α+β) of cellulose	Cell C6 (R+ia)	60.9	3.53	Kim & Ralph, 2014
C6/H6 (β) of internal and reducing ends (α+β) of cellulose	Cell C6 (R+ib)	60.7	3.80	Kim & Ralph, 2014
Hemicellulose (xylan)				
C3/H3 of (1→2) and (1→3)-α-L-arabinofuranose in xylans and xyloglucans	α-L-Araf C3	77.8<br>77.5<br>77.0	4.00<br>3.89<br>3.98	Komatsu & Kikuchi, 2013
C4/H4 of internal 4-O-methyl-α-D-glucuronic acid linked O-2 to (1→4)-α-D-xylopyranose	Xmga C4 (i)	77.8	3.65	Soucémarianadin et al., 2017
C3/H3 of non-reducing end of xylan	Xyl C3 (NR)	77.1	3.06	Kim & Ralph, 2014
C4/H4 of xylan internal & reducing end (α+β)	Xyl C4 (R+i)	75.9	3.50	Soucémarianadin et al., 2017
C4/H4 of 3-O-acetylated xylopyranose	Ac-Xylp C4	75.9	3.82	De Moura Neto et al., 2011
C2/H2 of reducing end (β) of xylan	Xyl C2 (R)	75.2	2.90	Kim & Ralph, 2014
C3/H3 of internal xylan	Xyl C3 (i)	73.5	3.29	Soucémarianadin et al., 2017
C2/H2 of xylan, internal & non-reducing end	Xyl C2 (NR+i)	72.5	3.17	Kim & Ralph, 2014
C3/H3 of β-D-galactopyranose	β-D-Galp C3	72.0	3.56	Soucémarianadin et al., 2017
C3/H3 of reducing end (α) of xylan	Xyl C3 (R)	71.2	3.62	Kim & Ralph, 2014
C2/H2 of β-D-galactopyranose	β-D-Galp C2	70.1	3.57	Soucémarianadin et al., 2017
C4/H4 of non-reducing end of xylan	Xyl C4 (NR)	70.0	3.42	Soucémarianadin et al., 2017
C2/H2 of 3-O-acetylated mannopyranose	Ac-Manp C2	69.4	3.70	De Moura Neto et al., 2011
C5/H5 of xylan internal & reducing end (β)	Xyl C5 (R+ib)	63.4	3.78	Soucémarianadin et al., 2017
C5/H5 of internal xylan	Xyl C5 (ia)	63.4	3.24	Soucémarianadin et al., 2017
C5/H5 (1→6)-α-L-xylopyranose in xyloglucan	α-L-Xylp C5	62.5	3.56	Komatsu & Kikuchi, 2013
C5/H5 of (1→2) and (1→3)-α-L-arabinofuranose in xylans and xyloglucans	α-L-Araf C5	62.1<br>61.9	3.51<br>3.59	Soucémarianadin et al., 2017
Lignin, Cutin and Suberin				
β-aryl ether linkages of lignin units (Cα/Hα)	β-O-4' (α)	71.8	5.02	Kim & Ralph, 2014
Methoxy group of the lignin units	Methoxy	56.2	3.74	Soucémarianadin et al., 2017
Phenylcoumaran (Cβ/Hβ) of lignin	β-5	52.0	3.62	Soucémarianadin et al., 2017
Methine group α in mid-chain alcohols	R-$\mathbf{C_{\alpha}H}$OH-R'	71.5	3.39	Soucémarianadin et al., 2017
Methylene units α to oxygen of an ester	R-$\mathbf{C_{\alpha}H_2}$-OCOR'	64.0	3.97	Soucémarianadin et al., 2017
Methylene α in a primary alcohol chain	R-$\mathbf{C_{\alpha}H_2}$-OH	61.3	3.36	Soucémarianadin et al., 2017
**Anomeric C region**				
C1/H1 of (1→4)-β-D-galactopyranose	β-D-Galp C1	105.4	4.35	Kim & Ralph, 2010
C1/H1 of (1→4)-β-D-mannopyranose	β-D-Manp C1	101.0	4.54	Soucémarianadin et al., 2017
C1/H1 of α-L-fucopyranosyl	α-L-Fucop C1	100.4	5.07	Soucémarianadin et al., 2017
C1/H1 of reducing-end of (1→4)-β-D-galactopyranose & (1→4)-β-D-xylopyranose	β-D-Galp(R) +β-D-Xylp(R)	97.8	4.23	Soucémarianadin et al., 2017
C1/H1 of reducing-end of (1→4)-α-D-galactopyranose & (1→4)-α-D-xylopyranose	α-D-Galp(R) +α-D-Xylp(R)	92.8	4.90	Soucémarianadin et al., 2017

C1/H1 of reducing-end of (1→4)-α-D-glucopyranose & (1→4)-α-D-xylopyranose	α-D-Gluc(R) +α-D-Xylp(R)	92.3	5.18	Komatsu & Kikuchi, 2013
Cellulose				
C1/H1 of non-reducing end of cellulose	Cell C1 (NR)	103.7	4.24	Soucémarianadin et al., 2017
C1/H1 of internal cellulose	Cell C1 (i)	103.3	4.32	Soucémarianadin et al., 2017
Hemicellulose (xylan)				
C1/H1 of α-arabinofuranosyl residues with glycosidic linkages to xylopyranose units	α-L-Araf C1	108.5	4.77	Soucémarianadin et al., 2017
C1/H1 of xylan, internal & non-reducing end	Xyl C1 (NR+i)	102.4	4.25	Kim & Ralph, 2014
C1/H1 of 2-O-acetylated xylopyranose	2-O-Xylp C1	99.6	4.64	Soucémarianadin et al., 2017
**Aromatic C region**				
Vinylic groups from suberin	vinyl (S)	130.1	5.32	Soucémarianadin et al., 2017
C2/C6 of p-hydroxyphenyl unit from lignin, suberin or tannin	*p*-H C2/C6	129.7<br>128.5	7.21<br>7.22	Soucémarianadin et al., 2017
C3/C5 of p-coumaroyl unit from lignin	*p*-CA C3/C5	115.8	6.77	Soucémarianadin et al., 2017
C3/C5 of p-coumaroyl unit from lignin or C5 of guaiacyl unit from lignin or suberin	*p*-CA C3/C5 or G-C5	115.4	6.64	Soucémarianadin et al., 2017

Table S2. Molecular moieties of cluster A', B' and C' from the PCA in Figure 3 of the main document.

Moiety	Abbreviation	δC (ppm)	δH (ppm)
**Cluster A'**			
C4/H4 of reducing end of (1→4) α or β-D-glucopyranose	D-Glup C4	82.5	3.54
C3/H3 of (1→2) and (1→3)-α-L-arabinofuranose in xylans	α-L-Araf C3	77.8	4.00
		77.5	3.89
		77.0	3.98
C4/H4 of internal 4-O-methyl-α-D-glucuronic acid linked O-2 to (1→4)-α-D-xylopyranose	Xmga C4 (i)	77.8	3.65
C3/H3 of non-reducing end of xylan	Xyl C3 (NR)	77.1	3.06
C3/H3 of (1→2) and (1→3)-α-L-arabinofuranose in xylans	α-L-Araf C3	77.8	4.00
C4/H4 of 3-O-acetylated xylopyranose	Ac-Xylp C4	75.9	3.82
C2/H2 of reducing end (β) of xylan	Xyl C2 (R)	75.2	2.90
C3/H3 of (1→4) α-D-glucopyranose (starch)	Starch C3	74.9	3.79
C4/H4 of (1→4) α-D-glucopyranose (starch)	Starch C4	73.4	3.64
C5/H5 of (1→4) α-D-glucopyranose (starch)	Starch C5	73.3	3.45
C3/H3 of β-D-galactopyranose	β-D-Galp C3	72.0	3.56
C2/H2 of (1→4) α-D-glucopyranose (starch)	Starch C2	70.4	3.13
C2/H2 of β-D-galactopyranose	β-D-Galp C2	70.1	3.57
C5/H5 of xylan internal & reducing end (β)	Xyl C5 (R+ib)	63.4	3.78
C5/H5 of internal xylan	Xyl C5 (ia)	63.4	3.24
C5/H5 (1→6)-α-L-xylopyranose in xyloglucan	α-L-Xylp C5	62.5	3.56
C5/H5 of (1→2) and (1→3)-α-L-arabinofuranose	α-L-Araf C5	62.1	3.51
C1/H1 of α-arabinofuranosyl residues with glycosidic linkages to xylopyranose units	α-L-Araf C1	108.5	4.77
C1/H1 of reducing-end of (1→4)-α-D-glucopyranose (starch) & (1→4)-α-D-xylopyranose	α-D-Gluc(R) +α-D-Xylp(R)	92.3	5.18
O-acetyl groups of hemicelluloses	R-COO**CH_3**	21.5	1.91
		21.4	2.02
Methyl groups adjacent to carb. acid or carbonyl/oxygen of an ester	**CH_3**-CHR-COOR'	17.0	1.09
Phenylcoumaran (Cβ/Hβ) of lignin	β-5	52.0	3.62
**Cluster B'**			
C4/H4 of internal and reducing ends (α+β) of cellulose	Cell C4 (R+i)	80.9	3.33
C5/H5 and C3/H3 of internal & non-reducing end of cellulose	Cell C5+C3 (NR+i)	76.9	3.14
C3/H3 of internal & reducing end (β) of cellulose	Cell C3 (R+i)	75.2	3.36
C2/H2 of reducing end (β) of cellulose	Cell C2 (R)	74.7	3.00
C2/H2 of internal cellulose	Cell C2 (i)	73.4	3.06
C4/H4 of non-reducing end of cellulose	Cell C4 (NR)	70.2	3.27
C5/H5 of reducing end (α) of cellulose	Cell C5 (R)	70.1	3.77
C6/H6 (a) of internal and reducing ends (α+β) of cellulose	Cell C6 (R+ia)	60.9	3.53
C6/H6 (b) of internal and reducing ends (α+β) of cellulose	Cell C6 (R+ib)	60.7	3.80
C1/H1 of non-reducing end of cellulose	Cell C1 (NR)	103.7	4.24
C1/H1 of internal cellulose	Cell C1 (i)	103.3	4.32
Methyl groups adjacent to carb. acid or carbonyl/oxygen of an ester	**CH_3**-CHR-COOR'	18.3	1.13
Methine group α in mid-chain alcohols	R-**$C_\alpha H$**OH-R'	71.5	3.39
C4/H4 of xylan internal & reducing end (α+β)	Xyl C4 (R+i)	75.9	3.50
C3/H3 of internal xylan	Xyl C3 (i)	73.5	3.29
C2/H2 of xylan, internal & non-reducing end	Xyl C2 (NR+i)	72.5	3.17
C3/H3 of reducing end (α) of xylan	Xyl C3 (R)	71.2	3.62
C4/H4 of non-reducing end of xylan	Xyl C4 (NR)	70.0	3.42
C2/H2 of 3-O-acetylated mannopyranose	Ac-Manp C2	69.4	3.70

C1/H1 of (1→4)-β-D-galactopyranose	β-D-Galp C1	105.4	4.35
C1/H1 of xylan, internal & non-reducing end	Xyl C1 (NR+i)	102.4	4.25
C1/H1 of (1→4)-β-D-mannopyranose	β-D-Manp C1	101.0	4.54
C1/H1 of reducing-end of (1→4)-β-D-galactopyranose & (1→4)-β-D-xylopyranose	β-D-Galp(R) +β-D-Xylp(R)	97.8	4.23
**Cluster C'**			
Methylene in aliphatic chains **α** to carb. acid or carbonyl of an ester	R-$\mathbf{C_\alpha H_2}$-COOR'	34.1	2.18
		34.0	2.26
Methylene in aliphatic chains **β** to carb. acid or carbonyl of an ester	R-$\mathbf{C_\beta H_2}$-CH_2COOR'	24.9	1.48
Methylene **β** in a primary alcohol chain in waxes	R-$\mathbf{C_\beta H_2}$-CH_2OH	33.6	1.52
		32.9	1.39
Methylene and methine groups in aliphatic chains: polymethylene chains from lipids, fatty acids and waxes	$(CH_2)_n$ + CH	31.7	1.24
		29.4	1.23
		25.9	1.26
		24.8	1.25
		22.5	1.26
Terminal methyl groups of aliphatic chains	R-$(CH_2)_n$-$\mathbf{CH_3}$	14.4	0.85
Methyl groups in waxes	$\mathbf{CH_3}$ from waxes	11.5	0.79
Methylene units **β** to oxygen of an ester	R-$\mathbf{C_\beta H_2}$-CH_2OCOR'	28.7	1.52
Methylene units **α** adjacent to alkene	R-$\mathbf{C_\alpha H_2}$-CH=CHR'	27.0	1.98
N-acetyl groups from peptidoglycan	R-NHCO$\mathbf{CH_3}$	23.4	1.83
Methyl groups from amino acids in peptides/proteins, and branched methyl groups in lipids and fatty acids	$\mathbf{CH_3}$ from amino acids + lipids	23.0	0.84
		21.9	0.82
		22.1	0.99
		20.0	0.82
		19.9	1.03
		19.1	0.90
		18.5	0.82
		16.8	0.92
		15.8	0.80
		12.2	0.82
Methylene units α to oxygen of an ester	R-$\mathbf{C_\alpha H_2}$-OCOR'	64.0	3.97
Methylene α in a primary alcohol chain	R-$\mathbf{C_\alpha H_2}$-OH	61.3	3.36
Vinylic groups (S)	vinyl (S)	130.1	5.32
Methoxy group of the lignin units	Methoxy	56.2	3.74

SUPPLEMENTAL REFERENCES

De Moura Neto, E., da S. Maciel, J., Cunha, P.L.R., de Paula, R.C.M. & Feitosa, J.P.A. (2011). Preparation and characterization of a chemically sulfated cashew gum polysaccharide. *J. Braz. Chem. Soc.* 22(10): 1953-1960.

Deshmukh, A.P., Simpson, A.J. & Hatcher, P.G. (2003). Evidence for cross-linking in tomato cutin using HR-MAS NMR spectroscopy. *Phytochemistry* 64, 1163–1170.

Hertkorn, N., Permin, A., Perminova, I., Kovalevskii, D., Yudov, M., Petrosyan, V. & Kettrup, A. (2002). Comparative analysis of partial structures of a peat humic and fulvic acid using one- and two-dimensional nuclear magnetic resonance spectroscopy. *Journal of Environmental Quality* 31, 375–387.

Kelleher, B.P. & Simpson, A.J. (2006). Humic substances in soils: are they really chemically distinct? *Environ. Sci. Technol.* 40: 4605-4611.

Kim, H. & Ralph, J. (2010). Solution-state 2D NMR of ball-milled plant cell wall gels in DMSO-d6/pyridine-d5. *Organic & Biomolecular Chemistry* 8, 576–591.

Kim, H. & Ralph, J. (2014). A gel-state 2D-NMR method for plant cell wall profiling and analysis: a model study with the amorphous cellulose and xylan from ballmilled cotton linters. *RSC Advances* 4, 7549–7560.

Komatsu, T. & Kikuchi, J. (2013). Comprehensive Signal Assignment of ^{13}C-Labeled lignocellulose Using Multidimensional Solution NMR and ^{13}C Chemical Shift Comparison with Solid-State NMR. *Anal. Chem.* 85(18): 8857–8865.

Sahakyan, A.B., Vranken, W.F., Cavalli, A. & Vendruscolo, M. (2011). Structural-based prediction of methyl chemical shifts in proteins. J. Biomol. NMR 50: 331-346.

Simpson, A.J., Song, G., Smith, E., Lam, B., Novotny, E.H. & Hayes, M.H.B. (2007). Unraveling the structural components of soil humin by use of solution-state nuclear magnetic resonance spectroscopy. *Environ. Sci. & Technol.* 41, 876–883.

Soucémarianadin, L.N., Erhagen, B., Nilssson, M.B., Öquist, M.G., Immerzeel, P. & Schleucher J. (2017). Two dimensional NMR spectroscopy for molecular characterization of soil organic matter: Application to boreal soils and litter. *Organic Geochemistry* 113: 184-195.
